# Construction and internal–external validation of a machine learning-based risk prediction model for multidrug resistance in ICU patients with acute exacerbation of chronic obstructive pulmonary disease

**DOI:** 10.3389/fmed.2026.1806672

**Published:** 2026-05-07

**Authors:** Yu Gu, Weiming Xu, Jing Xu, Yan Sun, Jiang Xin, Minxuan Ma

**Affiliations:** 1 Department of Clinical Laboratory, Affiliated Hospital of Jiangsu University, Zhenjiang, Jiangsu, China; 2 Department of Clinical Pharmacy, Baoying People's Hospital, Baoying Clinical Medical College of Yangzhou University, Yangzhou, Jiangsu, China; 3 Department of Hospital-Acquired Infection Control, Affiliated Hospital of Jiangsu University, Zhenjiang, Jiangsu, China

**Keywords:** AECOPD, LightGBM, machine earning, multidrug resistance, prediction model

## Abstract

**Objective:**

This study aimed to create and validate a machine learning (ML) model for predicting the risk of multidrug-resistant (MDR) infection in critically ill patients with acute exacerbation of chronic obstructive pulmonary disease (AECOPD) admitted to the intensive care unit (ICU).

**Methods:**

Data from patients diagnosed with AECOPD were retrospectively extracted from the Medical Information Mart for Intensive Care-IV (MIMIC-IV) database. A total of 1,018 patients were split at a 7:3 ratio into a training set (*n* = 712) for model development and an internal validation set (*n* = 306). MDR occurrence served as the outcome. Features were selected with the Boruta algorithm and least absolute shrinkage and selection operator (LASSO). Five ML algorithms—XGBoost, support vector machine (SVM), LightGBM, CatBoost, and Gaussian naïve Bayes (NB)—were trained with 10-fold cross-validation. Model performance was evaluated by the area under the receiver operating characteristic curve (AUC), accuracy, sensitivity, specificity, F1 score, calibration plot, decision curve, and clinical impact curve. An external validation cohort of 324 AECOPD patients admitted to the Affiliated Hospital of Jiangsu University between January 2024 and October 2025 was collected. The best-performing model was interpreted with Shapley Additive exPlanations (SHAP) to clarify feature importance and decision logic, and a nomogram was constructed to increase readability.

**Results:**

Boruta identified SpO₂, RBC, hemoglobin, hematocrit, blood urea nitrogen, creatinine, Nbpd, and Nbpm as significant predictors. The LightGBM outperformed the other algorithms: in the internal validation set, it achieved 78.9% accuracy, 90.5% sensitivity, 87.3% specificity, 60.5% F1 score, and an AUC of 0.966 (95% CI 0.951–0.981); in the external validation set, it reached 74.4% accuracy, 71.1% sensitivity, 77.8% specificity, 58.7% F1 score, and an AUC of 0.926 (95% CI 0.895–0.958). SHAP analysis indicated that hematocrit and SpO₂ were the primary drivers of model decisions. Interactive and dynamic nomograms were successfully developed.

**Conclusion:**

Multidrug-resistant occurrence in AECOPD patients was associated with SpO₂, RBC, hemoglobin, hematocrit, blood urea nitrogen, creatinine, Nbpd, and Nbpm. The LightGBM model demonstrated good discriminative ability but limited sensitivity for detecting positive cases, offering potential value as a rule-out screening tool for MDR risk in critically ill AECOPD patients admitted to ICU.

## 1 Introduction

Chronic obstructive pulmonary disease (COPD) is a prevalent chronic respiratory disorder characterized by persistent and progressive airflow limitation (1). Global estimates indicate that approximately 600 million people have been affected, and the disease ranks as the third leading cause of death worldwide, with particularly high incidence rates and mortality rates reported in low- and middle-income countries (2). Acute exacerbation of COPD (AECOPD) is recognized as a major driver of clinical deterioration, increased hospitalizations, accelerated lung function decline, and elevated mortality (3).

In patients with AECOPD, multidrug-resistant (MDR) bacterial infection has become an increasingly serious problem (4). Previous studies reported that the pulmonary MDR infection rate in this population reached 14.10%. In a cohort of 857 mechanically ventilated patients with severe AECOPD, Nseir et al. (5) identified MDR organisms in the lower respiratory tract of 8% of the subjects; these isolates accounted for 24% of all positive cultures and were associated with a significant prolongation of the ICU stay and a doubling of in-hospital mortality, indicating that resistant pathogens constitute a major driver of poor prognosis. (6) conducted a prospective observational study of 60 hospitalized AECOPD patients and reported a sputum-culture positivity rate of 40%, among which MDR bacteria represented 33.3% of the positive isolates (13.3% of the entire cohort); the treatment failure rate was markedly greater in the MDR group (37.5% versus 10%, *p* < 0.05), suggesting that MDR bacteria are key risk factors for therapeutic failure and clinical deterioration (6). Dao et al. (7) performed a prospective investigation of 92 Vietnamese AECOPD patients with concomitant pneumonia and reported a high prevalence of MDR infection; resistant pathogens not only prolonged hospitalization but also increased the risk of respiratory failure, underscoring the need to integrate local resistance surveillance data into empirical therapy and initiate targeted antimicrobial treatment promptly to improve outcomes. MDR bacterial infection has further increased therapeutic difficulty, extended the length of stay, and significantly increased healthcare costs and mortality (8).

It is important to note that the MIMIC-IV database exclusively comprises ICU admissions. Consequently, the present model was derived from a critically ill population with AECOPD, and its transportability to patients managed in emergency departments or general medical wards—who may exhibit different severity profiles, comorbidity burdens, and MDR pathogen epidemiology—remains uncertain and requires dedicated prospective validation.

The development of a machine learning-based risk prediction model for MDR in AECOPD patients is considered clinically and epidemiologically important. Accurate, early estimation of individual MDR probabilities allows clinicians to tailor empirical antibiotic therapy, curtail unnecessary broad-spectrum exposure, and thereby limit the emergence and spread of resistant organisms. Nseir et al. (5) pioneered this field by deriving a multivariable logistic regression MDRO risk index, which subsequently served as a methodological reference. Leighton et al. (9) subsequently constructed and externally validated the “RESIST score” in a 396-patient hospitalized-AECOPD cohort; decision curve analysis demonstrated that implementation of the score substantially reduced inappropriate broad-spectrum prescriptions and provided the first quantitatively validated tool for precision antimicrobial stewardship in acute COPD exacerbations. Nevertheless, prior AECOPD-MDR prediction models were typically built on small, single-center samples with conventional logistic regression, yielding limited external transportability and suboptimal calibration. Therefore, the present study aimed to develop and internally–externally validate a machine learning model for MDR risk in patients with AECOPD. This approach is anticipated to improve therapeutic efficacy, enhance patient prognosis, and concurrently curtail healthcare expenditures and the societal medical burden.

## 2 Methods

### 2.1 Data sources

Data for model development were extracted from MIMIC-IV v3.0 (Medical Information Mart for Intensive Care), a publicly available database jointly established by the Massachusetts Institute of Technology (MIT) and Beth Israel Deaconess Medical Center (BIDMC), Harvard Medical School. Available information includes patient measurements, medication orders, diagnoses, procedures, treatments, and de-identified free-text clinical notes. One team member completed the required training and obtained access certification (ID 68821009) and performed all the data extraction. The MIMIC project operated under an institutional review board protocol; all personal identifiers were removed, and each subject was referenced by a random code. The external validation cohort was supplied by the Affiliated Hospital of Jiangsu University; the local ethics committee approved the study (approval number: KY2023K0602).

### 2.2 Inclusion and exclusion criteria

The inclusion criteria were as follows: (1) ICU admission with a primary diagnosis of AECOPD identified by ICD-9/ICD-10 codes; (2) age ≥ 18 years; and (3) first ICU stay. The exclusion criteria were as follows: (1) total hospital length of stay < 48 h; (2) respiratory cultures obtained within 24 h of admission; (3) receipt of broad-spectrum antibiotics active against MDR organisms (e.g., carbapenems, anti-MRSA agents) before culture sampling; (4) cystic fibrosis; (5) history of lung or other solid-organ transplantation; (6) active malignancy; (7) HIV infection; and (8) missing laboratory or key clinical variables. The application of these criteria yielded 1,018 AECOPD patients for the final analysis cohort.

### 2.3 Data extraction

The research team extracted and managed all the data with PostgreSQL 15 via structured query language (SQL). The retrieved variables included hospital admission number, age, sex, comorbid conditions, and laboratory measurements. The documented comorbidities included hypertension, diabetes mellitus, heart failure, and renal impairment. The laboratory indices included white-cell count, hemoglobin, platelet count, alanine aminotransferase, aspartate aminotransferase, sodium, potassium, chloride, blood urea nitrogen, and serum creatinine. The primary endpoint was the occurrence of MDR.

### 2.4 Definition of multidrug resistance

(1) Gram-negative bacilli (e.g., *Klebsiella pneumoniae*, *Pseudomonas aeruginosa*, *Acinetobacter baumannii*, and *Escherichia coli*) that are non-susceptible (resistant or intermediate) to at least three of the following five antimicrobial classes: third−/fourth-generation cephalosporins (ceftriaxone, ceftazidime), fluoroquinolones (levofloxacin, ciprofloxacin), aminoglycosides (gentamicin, amikacin), carbapenems (imipenem, meropenem), and piperacillin-tazobactam. (2) methicillin-resistant *Staphylococcus aureus* (MRSA) is directly classified as MDR (10).

### 2.5 Statistical analysis

**Figure 1:**
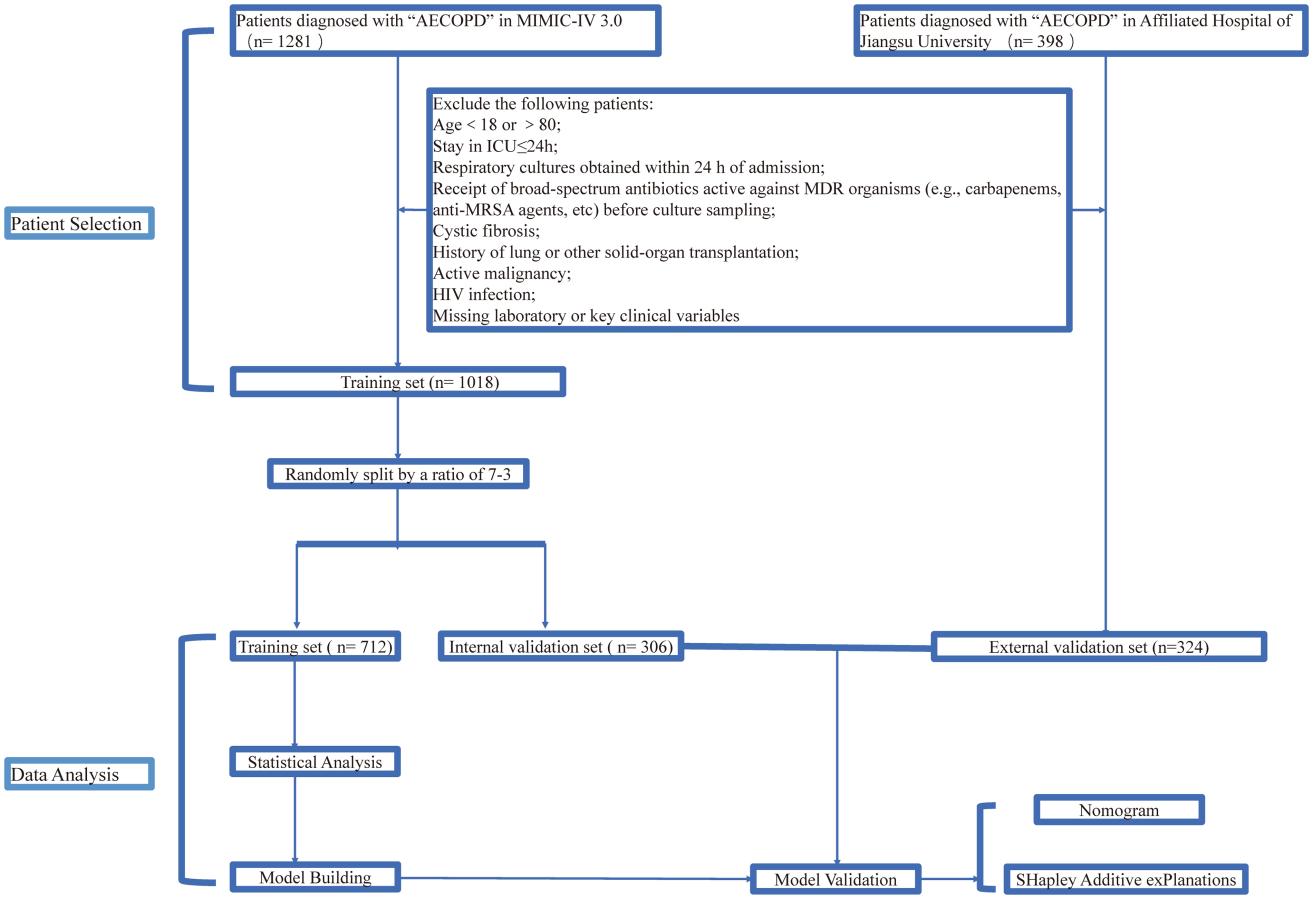
Flowchart of this study.

Patients were stratified into MDR and non-MDR groups according to the occurrence of MDR. Continuous variables are presented as the Mean ± Standard Deviation (SD) if normally distributed and were compared with Student’s t test; otherwise, they are expressed as medians (interquartile ranges, IQRs) and were evaluated with the Wilcoxon rank-sum test. Categorical variables are presented as percentages and were compared via the *χ*^2^ test. After excluding subjects with >5% missing data, multiple imputation was applied; no selected variable exceeded 30% missing data. The MIMIC cohort was randomly split 7:3 into a training set and an internal validation set. The training set underwent feature selection with the Boruta algorithm. Five machine learning algorithms—XGBoost, support vector machine (SVM), LightGBM, CatBoost, and Gaussian naïve Bayes (NB)—were trained on the selected features. Model performance was assessed in both the internal and external validation sets via the area under the receiver operating characteristic curve (AUC), sensitivity, specificity, recall, F1 score, accuracy, decision curve analysis (DCA), and calibration plots. The best-performing model was interpreted with Shapley Additive exPlanations (SHAPs) to quantify feature importance and decision logic, and a nomogram was constructed to increase clinical readability. All analyses were performed with R (v4.3.0) and STATA 17.0 (64-bit); two-sided *p*-values <0.05 were considered statistically significant. The study workflow is depicted in Figure 1.

## 3 Results

### 3.1 Baseline characteristics of the study population

After all patients were randomly allocated at a 7:3 ratio, 712 subjects constituted the training set, and 306 constituted the internal validation set. Between-group comparisons revealed a statistically significant difference only in Mi (*p* < 0.05), whereas all remaining variables showed no significant disparity (*p* > 0.05), indicating satisfactory randomization. Table 1 summarizes the comparative analysis of the baseline characteristics between the training and internal validation groups.

**Table 1:** Comparison between the training and internal-validation sets (*n* = 1,018).

Variables	Total (*n* = 1,018)	Test (*n* = 306)	Train (*n* = 712)	*P*
Age, mean ± SD	72.03 ± 11.08	72.40 ± 11.05	71.87 ± 11.10	0.491
Gender, *n* (%)				0.529
Female	511 (50.20)	149 (48.69)	362 (50.84)	
Male	507 (49.80)	157 (51.31)	350 (49.16)	
BMI, mean ± SD	29.60 ± 9.82	29.77 ± 10.02	29.52 ± 9.73	0.713
Wbc, mean ± SD	12.28 ± 6.79	12.39 ± 7.05	12.22 ± 6.68	0.717
Rbc, mean ± SD	3.74 ± 0.76	3.73 ± 0.78	3.74 ± 0.76	0.888
Platelet count, mean ± SD	219.38 ± 100.76	212.36 ± 95.53	222.39 ± 102.84	0.145
Hemoglobin, mean ± SD	11.04 ± 2.23	11.09 ± 2.29	11.01 ± 2.20	0.628
Rdw, mean ± SD	15.35 ± 2.20	15.41 ± 2.16	15.33 ± 2.21	0.589
Hematocrit, mean ± SD	34.58 ± 6.88	34.76 ± 7.24	34.50 ± 6.72	0.586
Sodium, mean ± SD	138.75 ± 5.11	138.97 ± 4.43	138.65 ± 5.37	0.368
Potassium, mean ± SD	4.40 ± 0.67	4.44 ± 0.75	4.38 ± 0.63	0.239
Calcium total, mean ± SD	8.50 ± 0.72	8.50 ± 0.71	8.51 ± 0.73	0.808
Chloride, mean ± SD	100.86 ± 6.54	100.76 ± 5.86	100.91 ± 6.82	0.733
Glucose, mean ± SD	150.95 ± 53.37	152.04 ± 53.44	150.48 ± 53.37	0.670
Anion gap, mean ± SD	13.73 ± 3.60	13.76 ± 3.42	13.72 ± 3.67	0.881
Ph, mean ± SD	7.35 ± 0.08	7.35 ± 0.08	7.35 ± 0.08	0.336
Pco2, mean ± SD	53.63 ± 14.72	54.02 ± 14.30	53.46 ± 14.90	0.575
Po2, mean ± SD	91.80 ± 55.36	88.67 ± 52.52	93.14 ± 56.52	0.237
Lactate, mean ± SD	1.75 ± 1.12	1.77 ± 1.08	1.74 ± 1.14	0.668
Totalco2, mean ± SD	30.02 ± 7.66	30.10 ± 7.29	29.99 ± 7.82	0.833
Pt, mean ± SD	15.91 ± 8.36	15.42 ± 6.44	16.13 ± 9.06	0.215
Ptt, mean ± SD	40.60 ± 24.57	39.99 ± 23.82	40.86 ± 24.90	0.605
Inr, mean ± SD	1.46 ± 0.80	1.42 ± 0.66	1.48 ± 0.85	0.265
Bilirubin total, mean ± SD	0.88 ± 1.73	0.95 ± 1.92	0.86 ± 1.65	0.466
Alt, mean ± SD	142.88 ± 491.56	134.52 ± 468.43	146.47 ± 501.45	0.722
Ast, mean ± SD	215.01 ± 869.94	190.54 ± 731.39	225.53 ± 923.41	0.557
Urea nitrogen, mean ± SD	29.91 ± 20.52	31.29 ± 21.76	29.32 ± 19.96	0.161
Creatinine, mean ± SD	1.34 ± 1.12	1.39 ± 1.26	1.32 ± 1.06	0.342
Hr, mean ± SD	86.99 ± 15.94	86.85 ± 15.87	87.04 ± 15.98	0.861
Nbps, mean ± SD	118.68 ± 16.42	118.00 ± 15.98	118.98 ± 16.61	0.381
Nbpd, mean ± SD	65.16 ± 14.88	65.47 ± 21.99	65.03 ± 10.44	0.663
Nbpm, mean ± SD	78.61 ± 11.66	77.98 ± 10.66	78.88 ± 12.06	0.255
Rr, mean ± SD	20.56 ± 3.73	20.86 ± 3.68	20.42 ± 3.75	0.084
Spo2, mean ± SD	95.38 ± 2.36	95.35 ± 2.34	95.40 ± 2.37	0.761
Temperature, mean ± SD	36.89 ± 2.21	36.85 ± 0.38	36.91 ± 2.64	0.685
Ht, *n* (%)	408 (40.08)	118 (38.56)	290 (40.73)	0.517
Dm, *n* (%)	334 (32.81)	99 (32.35)	235 (33.01)	0.839
Hf, *n* (%)	530 (52.06)	172 (56.21)	358 (50.28)	0.083
Mi, *n* (%)	107 (10.51)	41 (13.40)	66 (9.27)	0.049
Ckd, *n* (%)	233 (22.89)	71 (23.20)	162 (22.75)	0.876
Cirrhosis, *n* (%)	43 (4.22)	17 (5.56)	26 (3.65)	0.166
Hepatitis, *n* (%)	28 (2.75)	8 (2.61)	20 (2.81)	0.862
Pneumonia, *n* (%)	602 (59.14)	184 (60.13)	418 (58.71)	0.672
Stroke, *n* (%)	67 (6.58)	24 (7.84)	43 (6.04)	0.287
Hlp, *n* (%)	401 (39.39)	119 (38.89)	282 (39.61)	0.830
Aki, *n* (%)	823 (80.84)	246 (80.39)	577 (81.04)	0.810
Mdr, *n* (%)	120 (11.79)	35 (11.44)	85 (11.94)	0.820

IBM, Body Mass Index; Wbc, white blood cell; RBC, red blood cell; Rdw, red cell distribution width; Rdw, red cell distribution Width; PH, potential of hydrogen; PCO2, partial pressure of carbon dioxide; PO2, partial pressure of oxygen; Total CO₂, total carbon dioxide; PT, prothrombin time; PTT, partial thromboplastin time; INR, international normalized ratio; ALT, alanine aminotransferase; AST, aspartate aminotransferase; HR, heart rate; NBPD, non-invasive diastolic blood pressure; NBPS, non-invasive systolic blood pressure; NBPM, non-invasive mean arterial pressure; RR, respiratory rate; SPO2, saturation of peripheral oxygen; HT, hypertension; DM, diabetes mellitus; HF, heart failure; MI, myocardial infarction; HLP, hyperlipidemia; CKD, chronic kidney disease; AKI, acute kidney injury; MDR, multidrug resistant.

### 3.2 Feature selection with the Boruta algorithm

**Figure 2:**
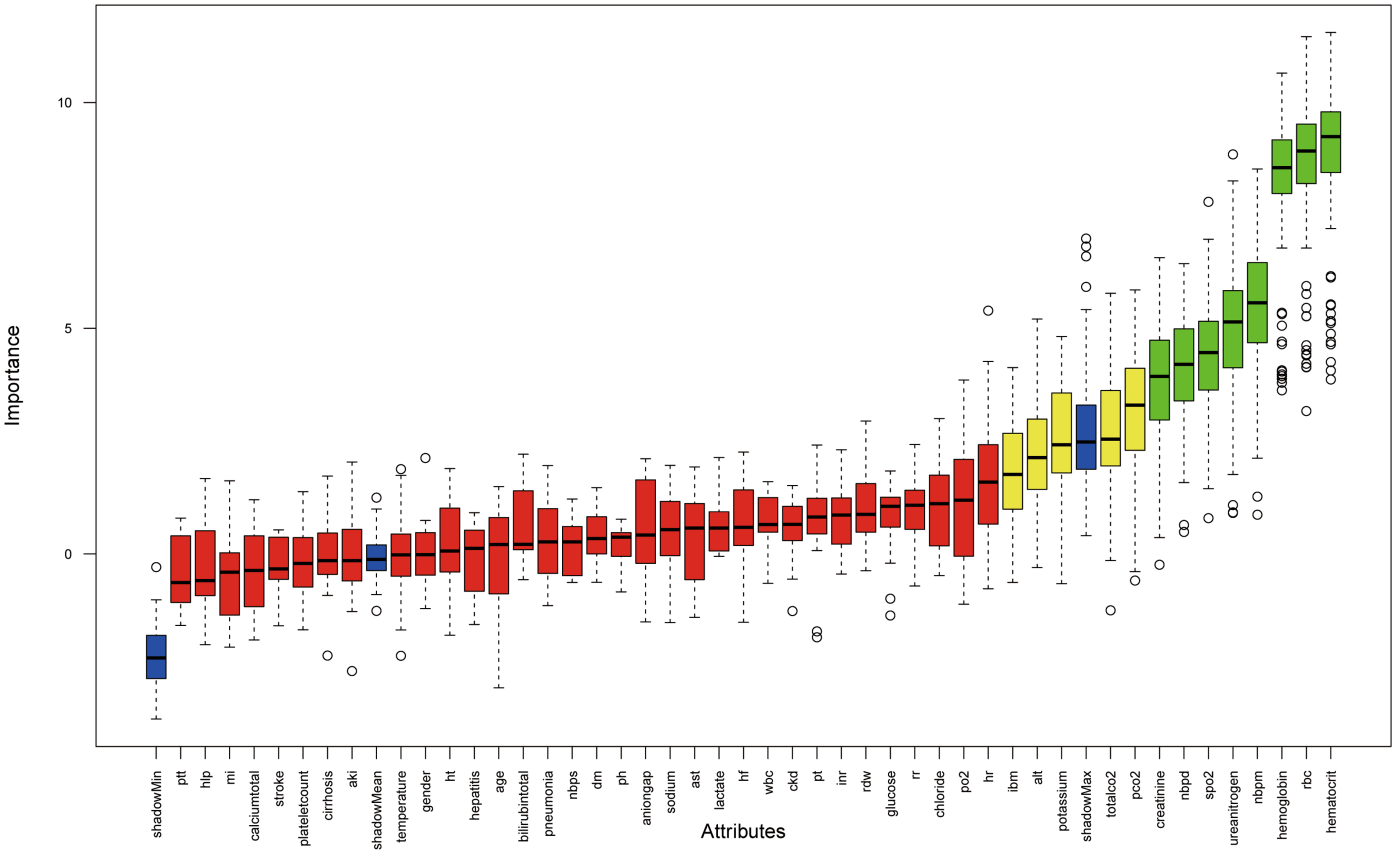
Boruta feature selection plot. The blue boxplots represent the minimum, mean, and maximum shadow (permuted) importance scores. Variables with green boxplots were considered important, yellow boxplots indicate tentative status, and red boxplots denote rejected features.

The Boruta algorithm, a random-forest-based wrapper for all-relevant feature selection, classifies variables into three categories: green (confirmed important), yellow (tentative), and red (unimportant). As illustrated in Figure 2, SPO₂, RBC, hemoglobin, hematocrit, blood urea nitrogen, creatinine, noninvasive diastolic blood pressure (Nbpd), and noninvasive mean arterial pressure (NBPM) were confirmed as important (green boxplots), whereas the remaining variables were either tentative or rejected. Consequently, these eight features were retained for subsequent machine learning model development.

### 3.3 Model construction and internal validation

**Figure 3:**
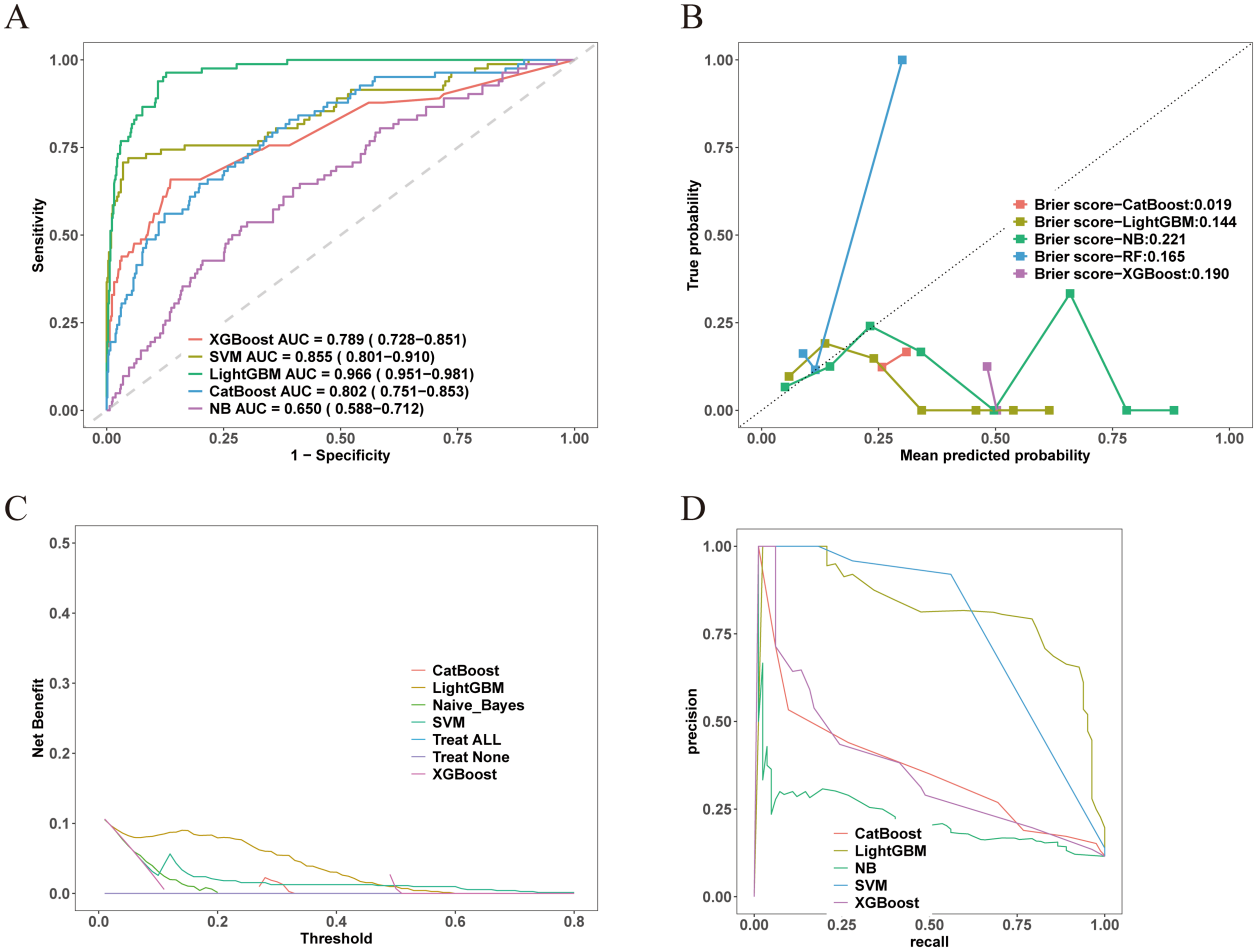
Performance comparison of the five machine learning models in the internal validation set: **(A)** ROC curves; **(B)** calibration curves; **(C)** decision curve analysis; **(D)** recall plot.

Using the eight predictors retained by Boruta, five machine learning algorithms—XGBoost, SVM, LightGBM, CatBoost, and naïve Bayes—were trained on the training set with grid-search hyperparameter tuning and 10-fold cross-validation to ensure stability. In the internal validation set, LightGBM achieved the highest discriminative performance, with an AUC of 0.966 (Figure 3A). The AUCs of the remaining models were 0.789 for XGBoost, 0.855 for SVM, 0.802 for CatBoost, and 0.650 for naïve Bayes. Calibration plots revealed that the predicted probabilities of LightGBM closely approximated the observed outcomes (*p* = 0.144), indicating good calibration (Figure 3B). Decision curve analysis revealed that XGBoost delivered stable net benefits across a wide range of threshold probabilities (Figure 3C). The detailed performance metrics are summarized in Table 2; LightGBM demonstrated the best overall performance (sensitivity 0.905, specificity 0.874, F1 score 0.605, accuracy 0.789) and the highest recall (0.905) (Figure 3D). Consequently, LightGBM was selected as the final optimal model.

**Table 2:** Performance of machine-learning models for predicting multidrug resistance.

Model	Set	AUC	Sensitivity	Specificity	Recall	F1	Accuracy
XGBoost	dev	0.799	0.744	0.646	0.744	0.333	0.695
XGBoost	vad	0.789	0.621	0.593	0.621	0.296	0.507
CatBoost	dev	0.853	0.659	0.803	0.659	0.415	0.731
CatBoost	vad	0.802	0.563	0.75	0.563	0.374	0.607
SVM	dev	0.899	0.768	0.968	0.768	0.764	0.868
SVM	vad	0.855	0.642	0.894	0.642	0.695	0.718
LightGBM	dev	0.982	0.939	0.933	0.939	0.766	0.936
LightGBM	vad	0.966	0.905	0.873	0.905	0.605	0.789
NB	dev	0.679	0.537	0.744	0.537	0.307	0.641
NB	vad	0.650	0.489	0.698	0.489	0.269	0.594

XGBoost, eXtreme Gradient Boosting; CatBoost, Categorical Boosting; SVM, Support Vector Machine; LightGBM, Light Gradient Boosting Machine; NB, Naive Bayes.

### 3.4 SHAP-based model interpretability analysis

**Figure 4:**
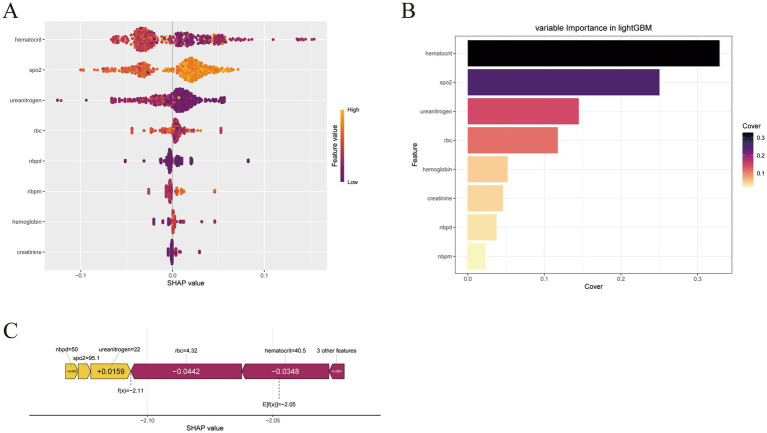
Interpretability analysis of logistic regression models. **(A)** SHAP dendrogram of features of the logistic regression model. **(B)** Importance ranking plot of features of the logistic regression model. **(C)** Interpretability analysis of 1 independent sample.

This study evaluated the relative importance of eight feature variables used to predict MDR in COPD patients. Figure 4A provides an intuitive ranking: each dot represents a single sample, and the blue-to-red gradient encodes the magnitude of the feature value. The vertical axis lists the features in order of importance, simultaneously displaying the correlation between feature values and SHAP values as well as their distributions. Figure 4B illustrates the directional influence of the eight variables on the prediction outcome; all eight variables contributed positively. It also presents the importance hierarchy within the logistic regression model, with features arranged along the vertical axis by rank and the horizontal axis indicating the mean SHAP values. To better understand the model’s decision-making at the individual level, we conducted a detailed interpretability analysis of a representative sample, as shown in Figure 4C. Visualizing the SHAP values for this instance clarified how each feature shaped the prediction for that specific case.

### 3.5 External validation of the machine-learning model

**Figure 5:**
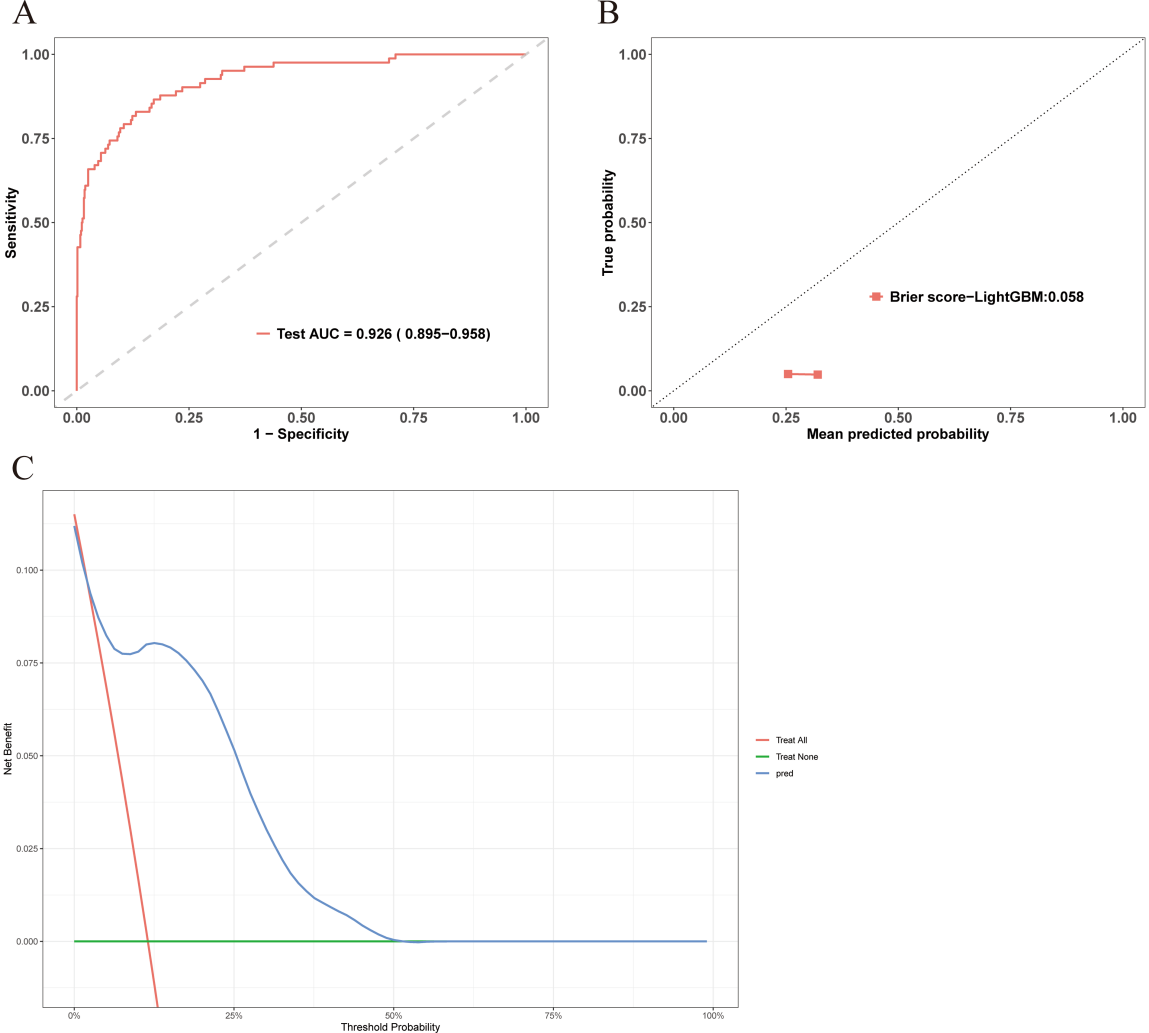
External validation of the XGBoost prediction model. **(A)** ROC curve in the external validation set. **(B)** Calibration curve in the external validation set. **(C)** Decision curve analysis in the external validation set.

The external cohort comprised 324 individuals; 308 (95.06%) were AECOPD patients without MDR, whereas 16 (4.94%) developed MDR. Baseline comparisons revealed no statistically significant differences between the two groups in RBC count, hemoglobin, hematocrit, systolic and diastolic noninvasive blood pressure, creatinine, blood urea nitrogen, or SpO₂ (all *p* > 0.05, Table 3). The ROC curve for the LightGBM model on the external validation set is shown in Figure 5A, yielding an AUC of 0.926 (95% CI 0.895–0.958), indicating good discriminative ability. However, the confusion matrix metrics in Table 4 reveal important limitations: the model demonstrated high specificity (0.961, 95% CI 0.934–0.988) and negative predictive value (0.954, 95% CI 0.925–0.983), but very low sensitivity (0.125, 95% CI 0.079–0.171), precision/PPV (0.142, 95% CI 0.094–0.192), and F1 score (0.133, 95% CI 0.080–0.190). These findings indicate that while the model accurately identifies patients who will not require invasive mechanical ventilation, it fails to detect the majority of true positive cases. Calibration plots demonstrated reasonable agreement between the predicted and observed probabilities (*p* = 0.058, Figure 5B), though the calibration slope of 0.891 and intercept of −0.132 suggest slight under-prediction of probabilities. Decision curve analysis further confirmed favorable net clinical benefits across a wide range of threshold probabilities (Figure 5C). Overall, the LightGBM model exhibited moderate external validity with notable trade-offs; it may serve as a useful screening tool to rule out the need for invasive mechanical ventilation in COPD patients complicated by respiratory failure, but its low sensitivity limits its utility as a standalone diagnostic instrument for identifying high-risk patients.

**Table 3:** Characteristics of the external validation cohort.

Variables	Total (*n* = 324)	No-MDR (*n* = 308)	MDR (*n* = 16)	*P*
Gender, *n* (%)				0.919
Female	166(51.23)	158(51.30)	8(50.00)	
Male	158(48.77)	150(48.70)	8(50.00)	
Rbc, mean ± SD	4.31 ± 0.62	4.31 ± 0.60	4.22 ± 0.88	0.686
Hemoglobin, mean ± SD	130.70 ± 19.70	130.85 ± 19.34	127.75 ± 26.31	0.648
Hematocrit, mean ± SD	40.58 ± 6.03	40.62 ± 5.93	39.65 ± 7.93	0.529
Nbps, mean ± SD	144.69 ± 23.64	145.27 ± 22.85	133.56 ± 34.72	0.201
Nbpd, mean ± SD	109.30 ± 38.94	109.46 ± 38.76	106.19 ± 43.56	0.744
Creatinine, mean ± SD	3.87 ± 3.98	3.81 ± 3.92	4.87 ± 5.07	0.301
Urea nitrogen, mean ± SD	4.05 ± 3.62	4.09 ± 3.64	3.17 ± 3.18	0.323
Spo2, mean ± SD	24.37 ± 30.85	24.54 ± 30.95	21.17 ± 29.53	0.671

RBC, red blood cell; NBPD, non-invasive diastolic blood pressure; NBPM, non-invasive mean arterial pressure; SPO2, saturation of peripheral oxygen; MDR, multidrug-resistant.

**Table 4:** Confusion matrix of the external validation set.

AUC	Sensitivity	Specificity	F1 score
0.926 (0.895–0.958)	0.125 (0.079–0.171)	0.961 (0.934–0.988)	0.133 (0.08–0.19)
Precision/PPV	NPV	Calibration intercept	Calibration slope
0.142 (0.094–0.192)	0.954 (0.925–0.983)	−0.132 (−0.230 to −0.034)	0.891 (−0.110, +0.086)

### 3.6 Construction of the nomograms

**Figure 6:**
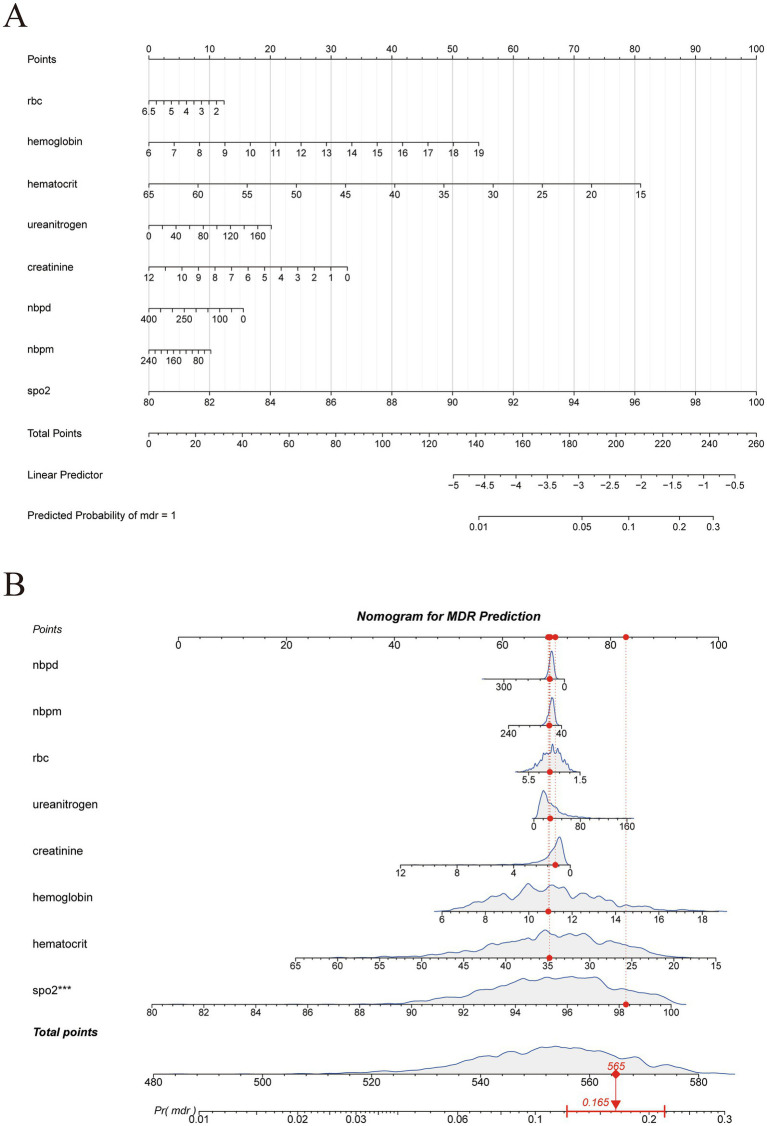
Construction of two distinct nomograms **(A,B)**.

Two nomograms were developed. Figure 6A integrates the eight key predictors—SpO₂, RBC count, hemoglobin, hematocrit, blood urea nitrogen, creatinine, diastolic noninvasive blood pressure (Nbpd), and mean noninvasive blood pressure (Nbpm)—to provide a visual estimate of the risk of MDR in COPD patients. Figure 6B presents an interactive nomogram; for the illustrated patient, a total score of 565 points corresponds to a 16.5% predicted probability of infection, offering a rapid and intuitively interpretable risk assessment.

## 4 Discussion

Chronic obstructive pulmonary disease, the third leading cause of death worldwide, is associated with acute exacerbation (AECOPD) complicated by multidrug-resistant (MDR) infections and has become a critical therapeutic challenge (11). At that time, clinicians lacked precise early warning tools for MDR infection control in AECOPD patients, and antibiotic overuse occurred frequently, further accelerating the spread and evolution of resistant organisms. Consequently, the development of efficient and reliable MDR risk prediction models is essential for individualized therapy and for optimizing antimicrobial stewardship, which has substantial clinical and public health value.

The present study drew on MIMIC-IV data from 1,018 AECOPD patients. Eight key predictors—SpO₂, red blood cell count (RBC), hemoglobin, hematocrit, blood urea nitrogen, creatinine, diastolic blood pressure (NBPD), and mean arterial pressure (NBPM)—were selected via the Boruta algorithm, and five machine learning models were constructed. The LightGBM achieved the best performance in internal validation, with an AUC of 0.966 and high sensitivity (0.905). External validation confirmed good discrimination (AUC 0.926) but revealed markedly reduced sensitivity (0.125), indicating that threshold-dependent metrics are highly susceptible to event-rate variability across settings. These findings align with LightGBM’s documented advantages in handling high-dimensional clinical data: its gradient-boosting decision-tree framework effectively captures nonlinear feature interactions, whereas histogram-based optimization and one-sided gradient sampling enhance training efficiency and generalizability—benefits that have been widely validated in critical-illness risk prediction (12).

SpO₂—especially when documented below 80% on admission—was positively associated with sputum culture positivity and subsequent multidrug-resistant (MDR) bacterial infection in AECOPD patients. Ramazan et al. (13) reported that among 240 hospitalized AECOPD patients in 2025, those presenting with SpO₂ < 80% presented an 80% sputum culture positivity rate, which was markedly higher than the 45.6% reported in patients with SpO₂ ≥ 80% (*p* < 0.001), suggesting that hypoxemia serves as a simple bedside indicator for bacterial infection and impending MDR risk.

No study has examined red blood cell (RBC) count per se as an independent predictor of MDR infection in patients with AECOPD. Nevertheless, elevated red-cell distribution width (RDW) and reduced hemoglobin have been linked to increased inflammatory burden (e.g., elevated CRP), prolonged length of stay, and more severe disease—settings in which MDR infection rates are known to rise (14, 15). Balasubramanian et al. (16), analyzing 2,539 stable COPD participants from COPDGene, described a U-shaped nonlinear relationship between hemoglobin and disease severity: anemic subjects experienced a 63% increase in hospitalization for severe exacerbations, implying that low hemoglobin—via a hypoxia–immunosuppression axis—impaired airway clearance. Among AECOPD patients, those admitted with Hb < 125 g/L displayed a significantly greater proportion of MDR bacterial infections. Long et al. (17), using the eICU-CRD database of 3,399 AECOPD cases, demonstrated that a high blood urea-nitrogen-to-creatinine ratio (BCR ≥ 22.78) not only increased in-hospital mortality by approximately 1.5–1.9-fold but also coincided with increased leukocyte counts and increased rates of sepsis and invasive ventilation, implying an increased risk of secondary MDR infection. Zhou et al. (18) prospectively followed 13,633 Chinese AECOPD patients and reported that admission diastolic blood pressure < 70 mmHg was independently associated with both a higher incidence of pneumonia and in-hospital death; however, MDR infection was not stratified, leaving direct evidence linking DBP or mean arterial pressure to MDR infection in AECOPD patients still lacking.

Among the patients enrolled in this study, females accounted for 50.2% (511/1018) and males accounted for 49.8% (507/1018), indicating a balanced gender distribution. Previous studies have suggested that the epidemiological characteristics of MDR infection in COPD patients may exhibit gender differences. Female COPD patients often demonstrate higher frequencies of acute exacerbations and distinct pathogen distribution patterns (19). Notably, our feature selection process (Boruta algorithm) did not identify gender as a critical variable for predicting MDR infection, which may be attributable to the balanced male-to-female ratio in our study sample. Alternatively, this finding may suggest that physiological indicators (such as SpO₂, hemoglobin, etc.) possess stronger predictive value than gender itself in risk prediction for AECOPD complicated by MDR infection. However, considering that gender may influence antibiotic usage patterns and exposure history to resistant bacteria, future studies should conduct stratified analyses of gender effects on model predictive performance in larger samples, and explore the necessity of gender-specific prediction models.

The SHAP analysis subsequently revealed the model’s decision logic (20) and identified hematocrit and SpO₂ as the dominant drivers of MDR-infection risk. Hematocrit, which denotes the fractional volume of erythrocytes, has been observed to deviate—either upward or downward—in response to hypoxia, inflammatory stress, and inadequate tissue perfusion in AECOPD (21). SpO₂, a direct surrogate of blood oxygen saturation, decreases whenever pulmonary ventilation or gas exchange deteriorates and thus constitutes a cardinal manifestation of AECOPD exacerbation (22). These findings align closely with established pathobiology: hypoxic milieus induce airway-epithelial injury, disrupt mucosal-barrier integrity, and thereby create niches favorable for resistant bacterial colonization and proliferation (23); concurrently, hypoxia-triggered systemic inflammation recruits immune cells that release abundant cytokines, amplifying tissue damage and compromising host defense (24). The additional inclusion of urea nitrogen and creatinine further implied that multiorgan crosstalk contributed to the evolution of MDR infection, which is consistent with prior reports that identified renal dysfunction as an independent predictor of infectious complications in critically ill patients (25).

Compared with traditional clinical-prediction rules, the LightGBM model constructed in this study demonstrated marked advantages. First, it relies solely on objective laboratory values and vital signs that are readily available and highly reproducible, thereby eliminating the bias inherent in subjective scoring systems. Second, internal validation demonstrated strong performance, while external validation confirmed good discrimination (AUC 0.926) but revealed substantial degradation in sensitivity (0.125) due to the low event rate (4.9%). These findings illustrate the classic ‘excellent in-house, disappointing elsewhere’ phenomenon and underscore the need for cautious interpretation of threshold-dependent metrics when event rates differ across settings. Finally, the interactive nomogram translated the complex algorithm into an intuitive risk score: clinicians could enter eight bedside variables to estimate the probability of MDR infection; however, given the low positive predictive value (14.2%) in the external cohort, the nomogram is best utilized to identify low-risk patients rather than to confirm high-risk status, furnishing a rapid and practical decision-support tool.

The clinical impact of the study revealed three lines: (1) by accurately flagging high-risk individuals, it enables tailored prevention–control strategies—early combination antibiotic therapy, enhanced airway care, and optimized respiratory support—thereby lowering infection incidence, and (2) it promotes rational antimicrobial use and curtails the indiscriminate prescription of broad-spectrum agents, aligning with the tiered-antibiotic stewardship policy. (3) The key predictors identified—particularly SpO₂ and hematocrit—offered mechanistic leads for future bench work, such as whether hypoxia-driven or viscosity-driven inflammatory or immune pathways modulate the acquisition of resistant pathogens.

Several limitations remain. First, the primary data source was retrospective; although stringent inclusion and exclusion criteria were applied to control for confounding factors, selection bias could not be ruled out. Second, variables such as previous antibiotic exposure and the severity of underlying comorbidities were not incorporated, potentially limiting predictive accuracy. Third, the external validation cohort originated from a single center with a low event rate (4.9%). While the AUC remained high, the very low sensitivity (12.5%) and positive predictive value (14.2%) indicate limited clinical utility for case detection in this setting. Multicenter, large-scale prospective validation with higher event rates is essential to assess true generalizability. Future work should expand the sample size, integrate additional clinical characteristics (e.g., genetic biomarkers, inflammatory mediators), and exploit deep learning architectures to increase performance. Prospective interventional trials are also warranted to determine whether management guided by the model reduces MDR infection rates and mortality in patients with AECOPD, thereby providing high-grade evidence for clinical translation. Fourth, and most critically, the model was derived from an ICU-specific cohort (MIMIC-IV) and externally validated in a single-center ICU cohort. While we have demonstrated internal–external validity within the ICU setting, the model’s applicability to AECOPD patients in emergency departments or general wards is unknown. These populations differ in illness severity, antibiotic exposure patterns, and local resistance ecology. We explicitly caution against extrapolating our findings to non-ICU settings without further dedicated validation. Finished, the external validation cohort’s low MDR event rate (16/324, 4.9%) led to wide confidence intervals for threshold-dependent metrics and markedly reduced sensitivity. This reflects the inherent statistical uncertainty when validating rare-event prediction models and precludes definitive claims of broad generalizability (26).

## 5 Conclusion

The present study developed an MDR-infection risk prediction model for AECOPD patients based on LightGBM and eight readily available clinical variables. The algorithm delivered discriminative and interpretable estimates with moderate external validity in critically ill ICU populations, offering clinicians an objective decision-support tool primarily for ruling out low-risk patients for ICU-admitted AECOPD patients. By refining diagnostic and therapeutic workflows within the ICU setting, the model shows potential for reducing unnecessary antibiotic exposure in low-risk patients and merits further prospective validation, particularly in settings with higher event rates and non-ICU environments, before broader clinical implementation. Following prospective validation in non-ICU settings.

## Data Availability

The original contributions presented in the study are included in the article/supplementary material, further inquiries can be directed to the corresponding authors.
